# Pre-Meal Whey Protein Alters Postprandial Insulinemia by Enhancing β-Cell Function and Reducing Insulin Clearance in T2D

**DOI:** 10.1210/clinem/dgad069

**Published:** 2023-02-03

**Authors:** Kieran Smith, Guy S Taylor, Mark Walker, Lise H Brunsgaard, Kelly A Bowden Davies, Emma J Stevenson, Daniel J West

**Affiliations:** Population Health Sciences Institute, Newcastle University, Newcastle upon Tyne NE1 7RU, UK; Human Nutrition and Exercise Research Centre, Newcastle University, Newcastle upon Tyne NE1 7RU, UK; Population Health Sciences Institute, Newcastle University, Newcastle upon Tyne NE1 7RU, UK; Human Nutrition and Exercise Research Centre, Newcastle University, Newcastle upon Tyne NE1 7RU, UK; Biosciences Institute, Newcastle University, Newcastle upon Tyne NE2 4HH, UK; Health and Performance Nutrition, Arla Foods Ingredients Group P/S, Viby J 8260, Denmark; Population Health Sciences Institute, Newcastle University, Newcastle upon Tyne NE1 7RU, UK; Sport and Exercise Sciences, Manchester Metropolitan University, Manchester M1 7EL, UK; Population Health Sciences Institute, Newcastle University, Newcastle upon Tyne NE1 7RU, UK; Human Nutrition and Exercise Research Centre, Newcastle University, Newcastle upon Tyne NE1 7RU, UK; Population Health Sciences Institute, Newcastle University, Newcastle upon Tyne NE1 7RU, UK; Human Nutrition and Exercise Research Centre, Newcastle University, Newcastle upon Tyne NE1 7RU, UK

**Keywords:** Postprandial hyperglycemia, GLP-1, type 2 diabetes, insulin clearance, incretin

## Abstract

**Context:**

Treatments that reduce postprandial glycemia (PPG) independent of stimulating insulin secretion are appealing for the management of type 2 diabetes (T2D). Consuming pre-meal whey protein (WP) reduces PPG by delaying gastric emptying and increasing plasma insulin concentrations. However, its effects on β-cell function and insulin kinetics remains unclear.

**Objective:**

To examine the PPG-regulatory effects of pre-meal WP by modeling insulin secretion rates (ISR), insulin clearance, and β-cell function.

**Methods:**

This was a single-blind, randomized, placebo-controlled, crossover design study in 18 adults with T2D (HbA_1c_, 56.7 ± 8.8 mmol/mol) who underwent 2 240-minute mixed-meal tolerance tests. Participants consumed WP (15 g protein) or placebo (0 g protein) 10 minutes before a mixed-macronutrient breakfast meal. PPG, pancreatic islet, and incretin hormones were measured throughout. ISR was calculated by C-peptide deconvolution. Estimates of insulin clearance and β-cell function were modeled from glucose, insulin, and ISR. Changes in PPG incremental area under the curve (iAUC; prespecified) and insulin clearance (post hoc) were measured.

**Results:**

β-cell function was 40% greater after WP (*P* = .001) and was accompanied with a −22% reduction in postprandial insulin clearance vs placebo (*P* < .0001). Both the peak change and PPG iAUC were reduced by WP (−1.5 mmol/L and −16%, respectively; both *P* < .05). Pre-meal WP augmented a 5.9-fold increase in glucagon and glucagon-like peptide 1 iAUC (both *P* < .0001), and a 1.5-fold increase in insulin iAUC (*P* < .001). Although the plasma insulin response was greater following WP, ISR was unaffected (*P* = .133).

**Conclusion:**

In adults with T2D, pre-meal WP reduced PPG by coordinating an enhancement in β-cell function with a reduction in insulin clearance. This enabled an efficient postprandial insulinemic profile to be achieved without requiring further β-cell stimulation.

Trial registry ISRCTN ID: ISRCTN17563146

**Website link**: www.isrctn.com/ISRCTN17563146

For most people with controlled type 2 diabetes (T2D), controlling postprandial glycemic (PPG) excursions are fundamental to achieving optimal glycemic control (1). Treatments that potentiate β-cell activity and the release of insulin are therapeutic options for the regulation of PPG. However, the repeated overstimulation of the β-cell may accelerate the loss of β-cell function and lead to a deterioration in glycemic control in the long run (2). Accordingly, interventions that regulate PPG without requiring or further stimulating insulin release are appealing.

Nutritional therapies are central to the care of T2D (3) and represent the greatest opportunity to optimize glycemic control in a cost-effective manner (4). Consuming whey protein (WP) prior to a meal is a novel dietary approach shown to regulate PPG excursions (5) and improve day-to-day glucose control in adults with T2D (6). The mechanisms by which pre-meal WP regulates PPG excursions are primarily due to the enhanced secretion of glucagon-like peptide 1 (GLP-1), delaying of gastric emptying, and elevated postprandial insulinemia (5). The effects of pre-meal WP on β-cell function and insulin output are, however, unclear, with previous assertions having been derived from plasma insulin responses. Indeed, drawing inferences of insulin secretory activity and β-cell function from plasma insulin concentrations is problematic since circulating plasma insulin reflects the balance of 2 distinct processes: the secretion of insulin from pancreatic β-cells and insulin's clearance by both hepatic and extra-hepatic tissues (7). By not accounting for insulin secretion and insulin clearance, using plasma insulin concentrations may miss the true metabolic effects of a given antihyperglycemic treatment and lead to erroneous conclusions (8).

It is well appreciated that major determinants of PPG include the rate of gastric emptying and the secretion of insulin (9), of which 50% to 70% of meal-derived insulin release is potentiated by the incretin peptides (10, 11). The importance of insulin clearance in determining the overall postprandial insulinemic and glycemic response is, however, less clear but is gaining scientific interest (7). After the ingestion of a meal, there is a coordinated decline in insulin clearance that is coupled with an increase in insulin secretion in healthy individuals. This produces an appropriate plasma insulin profile to overcome insulin resistance and maintain euglycemia (12). In people with T2D, however, the physiological decline in postprandial insulin extraction is impaired, and when combined with the underlying pathophysiology of β-cell dysfunction and insulin resistance, is reported to contribute to postprandial hyperglycemia (12) and declining glycemic control (13).

While much research has focused on the gastrointestinal responses following pre-meal WP supplementation, its effects on insulin kinetics and β-cell function remain unknown. We, therefore, examined the mechanisms by which pre-meal WP reduces PPG in people with T2D using a validated model of insulin kinetics and β-cell function. It was hypothesized that a pre-meal WP bolus would amplify the secretion of insulin, while modestly reducing the metabolic clearance rate of insulin (MCRi) (14), thereby reducing PPG excursions to a mixed-nutrient meal.

## Methods

### Ethical Approval

The presented data were collected as part of a wider study (6). This trial was approved by the local National Health Service Research Ethics Committee (18/NE/0372) in accordance with the Declaration of Helsinki and was registered at ISRCTN (ISRCTN17563146).

### Participant Recruitment and Eligibility

Individuals with T2D from the North East of England were recruited by study advertisements. Inclusion criteria were age 30 to 60 years, duration of T2D of ≥ 1 year, stable treatment with lifestyle and/or oral medications for ≥ 3 months, glycated hemoglobin (HbA_1c_) of < 80 mmol/mol (9.5%), stable body mass and a body mass index (BMI) of ≤ 40 kg/m^2^. Exclusion criteria were treatment in injectable glucose-lowering therapies, a history of gastrointestinal disease or a requirement for medications known to affect gastrointestinal function or appetite.

**Figure 1.**
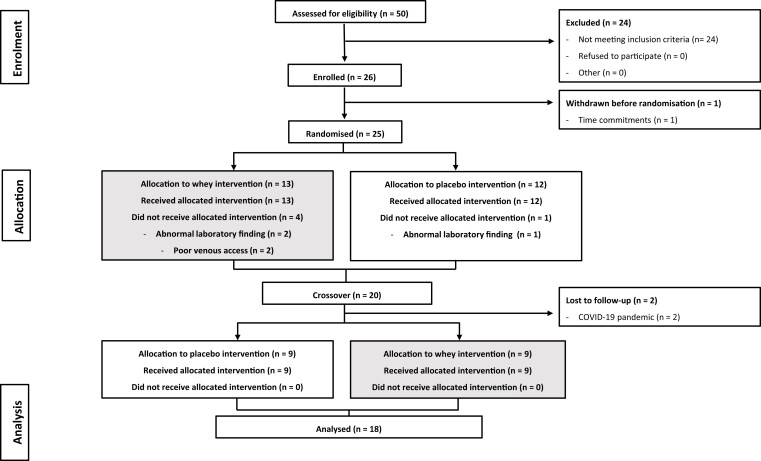
Participant flow through a randomized, placebo-controlled crossover trial.

Participant recruitment and testing were conducted between March 2019 and September 2021. A total of 26 participants were recruited for this study. From this cohort, 8 participants were withdrawn for the following reasons: 1 participant withdrew their consent prior to randomization; 3 participants were withdrawn due abnormal laboratory findings (laboratory measured HbA_1c_ > 80 mmol/mol); 2 participants were withdrawn due to the COVID-19 pandemic and the cessation of research activities; 2 participants were removed due to poor venous access (Fig. 1). Thus, the final study population consisted of 18 adults (female, n = 6) with T2D (HbA_1c_, 56.7 ± 8.8 mmol/mol [7.3 ± 0.8%]) treated by lifestyle modifications and/or oral medications with a self-reported diabetes duration of 5.7 ± 3.7 years (Table 1). All participants provided their written informed consent prior to enrollment into the study in accordance with Good Clinical Practice.

**Table 1.** Patient clinical characteristics

Characteristics (n = 18)	
Sex (male/female)	12/6
Age (years)	50 ± 5 (40-60)
Stature (cm)	171 ± 9.5
Body mass (kg)	95.8 ± 19.1 (61.9-130.5)
BMI (kg/m^2^)	32.7 ± 5.7 (21.3-43.6)
HbA_1c_ (mmol/mol)	56.7 ± 8.8 (42-73)
HbA_1c_ (%)	7.3 ± 0.8 (6.0-8.8)
Duration of diabetes (years)	5.7 ± 3.7 (2-20)
Family history of diabetes (n)	8
Diabetes treatment (n)	
− Metformin	5
− SU	1
− Metformin + SU	6
− Metformin + SGLT2i	3
− Metformin, SU + TZD	1
− Diet and lifestyle	2

Where appropriate, data are presented as means ± SD (range).

Abbreviations: SGLT2i, sodium-glucose cotransporter 2 inhibitor; SU, sulfonylureas; TZD, thiazolidinediones.

### Study Design

This was a single-blind, randomized, placebo-controlled, crossover study assessing the influence of pre-meal WP on parameters of glycemic control (6). Treatment sequences were determined by an online randomizer (https://www.randomizer.org/) and presented in a counterbalanced manner. Participants were blinded to their treatments. In brief, 2 laboratory-based feeding tests were performed with participants consuming a ready-to-drink WP (Lacprodan DI 6820; Arla Foods Ingredients Group P/S, Denmark) or placebo shot before a breakfast meal. Participants then consumed their pre-meal treatment over 7 days of free-living, with an interposed ∼14-day crossover period. Medications were kept stable and unaltered throughout. Data pertaining to the free-living component of the study have been published elsewhere (6).

### Procedures

Thirty-six hours before each mixed-meal test, participants were fitted with a continuous glucose monitoring system (Dexcom G6, Dexcom, Inc., USA) that was implanted into the subcutaneous tissue of the anterior-medial aspect of the abdomen (6). The Dexcom G6 measured interstitial glucose concentrations throughout the duration of the trial at a frequency of 5 minutes. This system is approved for the nonadjunctive management of diabetes and shows a high level of accuracy compared with point-of-care testing (15). The performance of strenuous bouts of physical activity and the consumption of alcohol were to be avoided 24 hours and 48 hours prior to each test, respectively. Dietary intake was standardized 24 hours prior to each feeding trial by completion of a food diary, which was replicated prior to the subsequent visit to ensure habitual dietary standardization.

Participants arrived at the Newcastle NIHR Clinical Research Facility after an overnight fast (∼12 hours), having consumed a standardized meal (920 kcal derived 47%, 17%, and 36% from carbohydrates, protein, and fat, respectively) the previous evening (∼1900-2000 hours). Once rested, an intravenous cannula was introduced into a forearm vein for repeated blood sampling. Participants then consumed a WP (100 kcal, 15.6 g protein) or placebo (35 kcal, < .1 g protein) shot 10 minutes before a breakfast meal consisting of 60 g of ready-to-eat cereal (Cheerios, Nestle, UK) and 250 mL of whole milk (387 kcal from 58% carbohydrates, 27% fat, and 15% protein). The breakfast meal was initiated at *t = −*0 minutes and consumed within 15 minutes. For 60 minutes following breakfast consumption, venous blood samples were collected at 15 minutes intervals, after which, samples were collected every 30 minutes for a further 180 minutes. Throughout the testing period participants remained seated while completing quiet activities such as reading, watching television, and typing.

### Pre-Meal Treatments

Patients were provided with 2 pre-meal shots created specifically for free-living glucose management (Arla Foods Ingredients Group P/S, Viby J, Denmark). Both the WP and placebo shots were presented as a 100 mL beverage in a ready-to-drink and contemporary format. Further details regarding to the pre-meal treatments and their acceptability in the free-living community are described elsewhere (6, 16).

### Sample Handling

For the analysis of C-peptide and insulin, blood samples were collected in serum collection tubes. For the analysis of glucagon, glucose-dependent insulinotropic polypeptide (GIP) and GLP-1, blood samples were collected in EDTA tubes containing aprotinin and a dipeptidyl-peptidase IV inhibitor. All collection tubes were centrifuged at 3000 rpm at 4 °C for 10 minutes and stored at −80 °C until analysis. PPG were measured by a Dexcom G6 continuous glucose monitoring system (Dexcom, Inc., USA).

### Calculations

The time-averaged incremental area under the curve (iAUC) (ie, the postprandial area above baseline concentrations [*t = −*15 minutes]) was calculated using the trapezoidal rule and was divided by the duration of interest. Prehepatic insulin secretion rates (ISR) were calculated from C-peptide deconvolution by utilizing a two-compartmental model of C-peptide kinetics and population-derived C-peptide parameters (17). Whole-body insulin sensitivity was calculated from the oral glucose insulin sensitivity index (OGIS) (18). The ratio of the iAUC_240min_ of ISR to PPG, corrected for insulin resistance (1/OGIS), was calculated as an integrated index of β-cell function (ΔISR/PPG÷IR]) (12). This index correlates well with β-cell glucose sensitivity calculated from a validated mathematical model of β-cell function (19).

A single-pool model was used to describe insulin kinetics, as previously reported (12, 20). In brief, fasting insulin clearance was calculated as ISR divided by plasma insulin concentration at time point *t = −*15 minutes. During the feeding test, the rate of insulin extraction [R_dins_(*t*)] (ie, hepatic and peripheral tissues) and metabolic clearance rate of insulin [MCRi(*t*)] at each time point during the trial (*t*) were calculated by:


$$\mathrm{Rdins}(t)=\mathrm{ISR}(t)\;\text{–}\;\left[\frac{\mathrm{dI}(t)}{\mathrm{dt}}\right]\times \mathrm{Vins}$$

$$\mathrm{MCRi}(t)=\frac{\mathrm{ISR}(t)}{\mathrm{Ins}(t)}-\frac{\left[\frac{\mathrm{dI}(t)}{\mathrm{dt}}\right]}{I(t)}\;\times \mathrm{Vins}$$

Where V_ins_ is the volume of distribution for insulin, which was estimated as 141 mL/kg (7). Total extraction and clearance of insulin during the mixed-meal tolerance test were calculated and time-averaged using:


AUCRdins=AUCISR–[Ins(t=240)–Ins(t=−15)]×Vins

$$\begin{array}{rl} \mathrm{AUC}\;\mathrm{MCRi}\;= & \;\mathrm{AUC}\;\left[\frac{\mathrm{ISR}(t)}{\mathrm{Ins}(t)}\right]\;\text{–}\;[\mathrm{Ln}\;(\mathrm{Ins}[t=240])\\ & \text{–}\;\mathrm{Ln}\;(\mathrm{Ins}[t=-15])]\times \;\mathrm{Vins} \end{array}$$

Where Ins[t = −15] and Ins[t = 240] are plasma insulin concentrations at baseline (*t = −*15 minutes) and at completion (*t* = 240 minutes) of the trial, respectively.

### Analytical Procedures

Serum insulin concentrations were determined using a commercially available enzyme-linked immunosorbent assay (ELISA) with an assay sensitivity of 6 pmol/L (intra-assay, < 8.9%; Cat# 10-1113-01, RRID:AB_2877672; Mercodia AB, Sweden). Serum C-peptide concentrations were measured using an ELISA with an assay sensitivity of < 25 pmol/L (intra-assay, < 10%; Cat# 10-1136-01, RRID:AB_2750847; Mercodia AB, Sweden). Plasma glucagon concentrations were measured using a sandwich ELISA employing monoclonal antibodies against the C- and N- terminal regions of glucagon with a detection limit of 0.75 pmol/L (intra-assay, < 4.3%; inter-assay, 9.0%; Cat 10-1271-01, RRID:AB_2737304; Mercodia AB, Sweden). Plasma GIP concentrations (ie, [1-42] and [3-42]) were analyzed by ELISA with an assay sensitivity of 1 pmol/L (intra-assay, < 5.4%; inter-assay, 10%; Cat# EZHGIP-54K, RRID:AB_2801401; Merck Millipore, USA). Plasma GLP-1 (ie, [7-36] NH_2_ and [9-39] NH_2_) concentrations were quantified using an ELISA with a sensitivity of 1.5 pmol/L (intra-assay, < 6%; inter-assay, 13%; Cat# EZGLP1T-36K, RRID:AB_2813786; Merk Millipore, USA).

### Statistical Analysis

All data were assessed for normal distribution by a Shapiro-Wilks test. The data that were revealed to be not normally distributed were log_10_ transformed and re-assessed for distribution. Where transformation failed, data were assessed non-parametrically. Time-response variables were assessed by a two-way repeated measures ANOVA or by a Friedman's test if the data presented as parametric or nonparametric, respectively. Post hoc analyses, adjusted for multiple comparisons using Bonferroni corrections, were performed where appropriate. Parametric and nonparametric data requiring a single comparison were analyzed by a paired sampled *t* tests or Wilcoxon Signed Ranks test, respectively. Bivariate correlational analysis was performed to detect the strength and direction of relationships between metabolites of interest. Unless stated otherwise, all data presented in text and in tables are presented as means ± SD or as median (interquartile range [IQR]) for excessively skewed data. Graphical data is expressed as means with 95% CI. Treatment differences (ie, WP—placebo) are presented as means (95% CI), or as the ratio of the geometric means (95% CI) for data that required transformation. A ratio of 1 indicates equal means between treatments, whereas a value of < 1 indicates a lower mean for WP compared to placebo. Statistics were performed using SPSS Statistics (v28; IBM Corp, USA) and Prism (v9.0; GraphPad Software, USA) and significance was accepted as *P* < .05.

## Results

Fasting biochemical variables were similar between trials (Table 2). Both pre-meal supplements were well tolerated. There were no adverse events or episodes of hypoglycemia during the feeding test or in the following 24 hours.

**Table 2.** Postprandial metabolic and modeling variables following consumption of a pre-meal WP or placebo shot

Variable	WP	Placebo	*P* value
Glucose			
Fasting (mmol/L)	8.1 ± 1.7	8.2 ± 1.6	.717
ΔPPG (mmol/L)	4.3 ± 2.0	5.8 ± 2.3^a^	**.013**
iAUC_60min_ (mmol/L/min^−1^)	1.8 (1.1, 2.7)	3.0 (2.1, 4.0)^a^	**.001**
iAUC_240min_ (mmol/L/min^−1^)	2.3 ± 1.1	2.7 ± 1.0^a^	**.005**
Insulin			
Fasting (pmol/L)	79.3 ± 48.2	83.6 ± 57.9	.605
iAUC_60min_ (pmol/L/min^−1^)	311.8 ± 187.6	221.6 ± 140.9^a^	**.001**
iAUC_240min_ (pmol/L/min^−1^)	211.1 ± 106.7	160.8 ± 113.8^a^	**<.001**
C-peptide			
Fasting (pmol/L)	911 ± 413.2	855.7 ± 430.4	.297
iAUC_60min_ (pmol/L/min^−1^)	929.0 ± 374.5	801.5 ± 416.0	.064
iAUC_240min_ (pmol/L/min^−1^)	1073.1 (904.2, 1163.1)	950 (820.9, 1114.7)	.107
ISR			
Fasting (pmol/min^−1^/m^−2^)	118.5 ± 53	110.5 ± 55.8	.108
iAUC_60min_ (pmol/min^−1^/m^−2^)	182.3 ± 62.5	167.4 ± 78.2	.234
iAUC_240min_ (pmol/min^−1^/m^−2^)	134.5 (125.3, 161.1)	124 (109.7, 151.2)	.133
GIP			
Fasting (pmol/L)	16.5 ± 9.6	15.9 ± 7.8	.711
iAUC_60min_ (pmol/L/min^−1^)	63.0 ± 25.3	64.4 ± 28.0	.656
iAUC_240min_ (pmol/L/min^−1^)	49.6 ± 15.8	42.2 ± 15.7^a^	**.014**
GLP-1			
Fasting (pmol/L)	32.5 ± 12.8	34.1 ± 14.2	.523
iAUC_60min_ (pmol/L/min^−1^)	25.6 ± 10.0	9.8 ± 6.0^a^	**<.0001**
iAUC_240min_ (pmol/L/min^−1^)	14.6 ± 6.7	4.4 ± 3.1^a^	**<.0001**
Glucagon			
Fasting (pmol/L)	15.4 ± 7.2	15.0 ± 7.1	.837
iAUC_60min_ (pmol/L/min^−1^)	21.2 ± 11.4	7.6 ± 6.1^a^	**<.0001**
iAUC_240min_ (pmol/L/min^−1^)	6.0 (4.6, 9.7)	1.6 (0.5, 7.0)^a^	**.001**
Modeling variables			
Fasting MCRi (L/min/m^−2^)	1.5 ± 0.5	1.4 ± 0.4	.109
MCR_i_ AUC (L/min/m^−2^)	1.07 ± 0.45	1.38 ± 0.67^a^	**<.0001**
Rd_ins_ AUC (pmol/min/m^−2^)	319. 7 ± 170.6	293.7 ± 80.8	.473
OGIS (mL/min^−1^/m^−2^)	256.8 ± 42.4	264.1 ± 40.5	.446
Log_10_ ΔISR/PPG÷IR	1.78 ± 0.29	1.64 ± 0.30^a^	**.001**

Data are presented as means ± SD or as median (IQR) for excessively skewed data. Analysis was conducted on all participants (n = 18) by a paired sampled *t* test or a Wilcoxon Signed Ranks test, where appropriate.

Abbreviations: GIP, glucose-dependent insulinotropic polypeptide; GLP-1, glucagon-like peptide 1; iAUC, incremental area under the curve; ISR, insulin secretion rate; MCRi, metabolic clearance rate of insulin; OGIS, oral glucose insulin sensitivity index; PPG, postprandial glycemia; Rdins(t), rate of insulin extraction.

and **boldface** denotes a treatment effect (*P* < .05).

### Glucose

**Figure 2.**
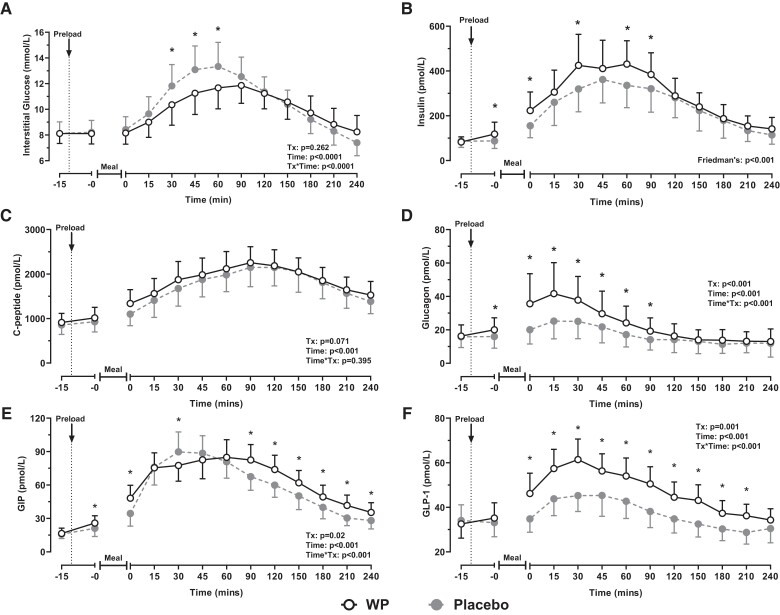
Postprandial profiles (means with 95% CI) of interstitial glucose (A) insulin (B), C-peptide (C), glucagon (D), GIP (E), and GLP-1 (F) following ingestion of a WP (open) or a placebo (filled) preload shot. Preloads were consumed 10 minutes prior to a mixed-nutrient meal. Data were analyzed by a two-way repeated measures ANOVA (panels a, c, d, e, and f) or a Friedman's ranks test (panel b). Post hoc pairwise comparisons were performed with Bonferroni corrections. Results from the ANOVA are presented by treatment (Tx), time, and differences due to the interaction of treatment*time (Tx*time). *Denotes a treatment effect (*P* < .05). For clarity, significant time effects are not highlighted in the figure.

Interstitial PPG concentrations increased following the ingestion of the mixed-nutrient breakfast, peaking at ∼*t* = 60 to 90 minutes before returning to preprandial values (Fig. 2A). Compared with the placebo trial, PPG concentrations were significantly lower from *t* = 30 to 60 minutes after the WP preload (time*treatment, *P* < .0001; all *P* < .021). Accordingly, the time-averaged PPG iAUC_240min_ was reduced by −16.4% (95% CI, −25.7 to −7.0%; *P* = .004) with WP. The incremental change in glycemia from basal to peak values (ΔPPG) was also −1.5 mmol/L (95% CI, −2.5 to −0.5 mmol/L; *P* = .013) lower following the WP pre-meal treatment (Table 2).

### Insulin and C-peptide

The ingestion of the meal caused plasma insulin and C-peptide to increase from baseline values (*P* < .001; Fig. 2B and 2C). However, only plasma insulin responses were affected by the pre-meal WP treatment, such that compared with the placebo intervention, insulin concentrations were greater immediately pre- and post- meal following the WP shot (*P* = .015 and *P* = .010, respectively), and remained elevated from *t* = 30 minutes (*P* = .002), and *t* = 60 to 90 minutes (both *P* = .020; Fig. 2B). Overall insulin iAUC_240min_ was approximately 1.5-fold greater during WP (*P* < .001; Table 2). In contrast, temporal responses for C-peptide were no different between trials (time*treatment, *P* = .395 [Fig. 2C]; iAUC_240min_, *P* = .107 [Table 2]).

### Glucagon

Plasma glucagon concentrations demonstrated significant main effects for both *time* (*P* < .001) and *treatment* (*P* < .001), such that glucagon concentrations increased from baseline following ingestion of the meal before returning to preprandial values (Fig. 2D). During the WP intervention, plasma glucagon concentrations remained elevated above baseline values from *t = −*0 to 60 minutes (all *P* < .017) before reaching their nadir, whereas during placebo, glucagon remained elevated from *t* = 15 to 30 minutes (both *P* < .024). A significant time*treatment interaction was also established (*P* < .001), revealing that plasma glucagon concentrations were greater during the WP trial from *t = −*0 to 90 minutes, compared with placebo (all *P* < .007). As such, WP increased the time-averaged glucagon iAUC by 5.9-fold during the early (0-60 minutes) and full (0-240 minutes) postprandial period (*P* < .001; Table 2).

### Incretin Peptides

During both experimental days, GIP increased from basal values following consumption of the test meal and remained elevated throughout the postprandial period (*time*, *P* < .001). Postprandial GIP responses differed between trials (time*treatment, *P* < .001), such that during the WP intervention, plasma GIP concentrations were elevated during the immediate (*t* = −0 minutes and t = 0 minutes) and latter (t = 90 to 240 minutes) postprandial phases, compared with placebo (all *P* < .017). However, relative to placebo, GIP concentrations were lower at *t* = 30 minutes with WP (*P* = .022), consistent with delayed gastric emptying (Fig. 2E). During the early postprandial period, the time-averaged GIP iAUC_60min_ was similar between trials (*P* = .656). On the other hand, GIP iAUC_240min_ during the WP trial was ∼17% greater (*P* = .014; Table 2).

During both trials, GLP-1 concentrations increased from basal values following ingestion of the breakfast meal before returning to preprandial concentrations upon termination of the trial (*time, P* = .001; Fig. 2F). Temporal postprandial GLP-1 responses differed between interventions (time*treatment, *P* < .001), such GLP-1 concentrations were elevated from *t* = 0 to 210 minutes during WP, compared with placebo (all *P* < .002). Both GLP-1 iAUC_60min_ and iAUC_240min_ were 3.9-fold and 5.9-fold greater during WP, respectively (*P* < .0001; Table 2).

### Insulin Kinetics

**Figure 3.**
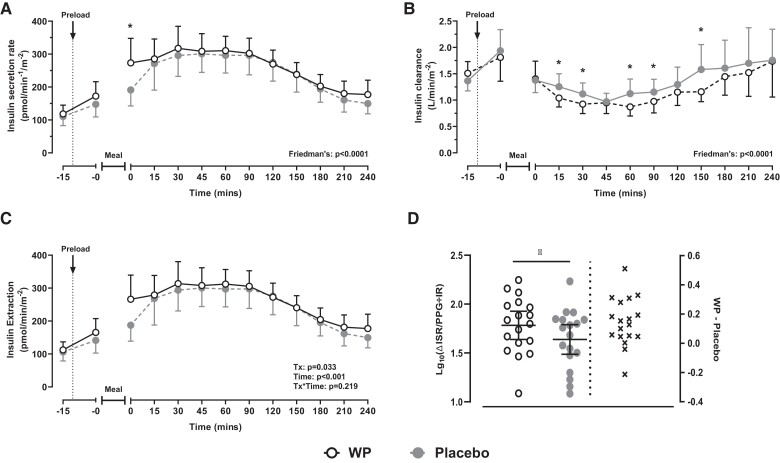
Postprandial profiles (means with 95% CI) of ISR (A) MCRi (B) and Rd_ins_ (C) following ingestion of a WP (open) or a placebo (filled) pre-meal shot 10 minutes prior to a mixed-nutrient meal. Time-responsive data were analyzed by a Friedman's test (panels a and b) or two-way repeated measures ANOVA (c). Post hoc pairwise comparisons were performed with Bonferroni corrections. Panel (d) reports changes in β-cell function (ie, ΔISR/PPG÷IR), in Log10 scale, as determined by a paired-samples *t* test. Results from the ANOVA are presented by treatment (Tx), time, and differences due to the interaction of treatment*time (Tx*time). *Denotes a treatment effect (*P* < .05). For clarity, significant time effects (panels a, b, and c) are not highlighted in the figure.

During both trials, ISR increased following the ingestion of the meal. Although WP potentiated an early increase in ISR, compared with placebo (*t* = 0 minutes [*P* = .016]; Fig. 3A), overall temporal ISR responses and iAUC_240min_ were similar during both study days (*P* = .133; Table 2).

Fasting MCRi values were similar at the commencement of both trials but differed following the meal (Fig. 3B). During the WP trial, MCRi declined from baseline from *t* = 15 to 150 minutes (all *P* < .004), whereas the decline in MCRi during the placebo intervention was less marked (*t* = 30 to 60 minutes; all *P* < .01). Compared with placebo, overall MCRi AUC was ∼22% lower during the WP intervention (ratio of the geometric means, 0.78 [95% CI, 0.73 to 0.84]; *P* <.0001). R_dins_, which is a product of MCRi and plasma insulin, showed main effects for both *time* (*P* < .001) and *treatment* (*P* = .033), but no interaction effects were observed (*P* = .219). R_dins_ increased following consumption of the meal and remained above baseline throughout the trial (Fig. 3C). Compared with placebo, R_dins_ was on average 17.1 pmol/min/m^2^ (95% CI, 1.5 to 32.6) greater during WP (*P* = .033), which likely reflects the increase in plasma insulin. Overall R_dins_ AUC was similar (*P* = .473).

### β-Cell Function and Whole-Body Insulin Sensitivity,

ΔISR/PPG÷IR was increased during the WP trial, relative to placebo (ratio of geometric means, 1.4 [95% CI, 1.16 to 1.69; *P* = .001]), indicating an improvement in β-cell function (Fig. 3D). Whole-body insulin sensitivity (OGIS) was unaffected by the pre-meal WP treatment (*P* = .446).

### Associations Between Insulin Secretion, Insulin Clearance, and Glycemia

Using pooled data, an inverse association between MCRi and glucagon iAUC_240min_ was revealed (*r_s_* = −0.393; *P* = .018). MCRi or PPG were not related to any other hormones.

## Discussion

The present study investigated the effects of an acute dose of pre-meal WP (15 g) on pancreatic β-cell function and postprandial insulin kinetics, and their subsequent influence on PPG excursions in people with T2D. Our primary finding is that, at the whole-body level, pre-meal WP integrates a reciprocal mechanism that involves an enhancement in β-cell function with a reduction in insulin clearance (MCRi). This produces an effective plasma insulin profile to rising glucose concentrations without requiring further β-cell stimulation and insulin release.

We confirm prior reports demonstrating that a low dose of pre-meal WP reduces PPG excursions in people with T2D (21–23). Our observed reduction in PPG was substantial (ie, ΔPPG, −1.5 mmol/L; iAUC_240min_, −16%) and was accompanied with the enhanced secretion of GIP, GLP-1, and glucagon, as well as a 2-fold increase in plasma insulin concentrations. However, by studying a true measure of insulin secretion (obtained by deconvolution of plasma C-peptide), our data provide new insights into postprandial insulin kinetics and pancreatic endocrine function. As shown from ΔISR/PPG÷IR, β-cell function was markedly improved during the WP intervention. Simply, the amount of insulin secreted and the responsiveness of its release relative to an incremental change in glucose was enhanced by pre-meal WP, thus demonstrating a more efficient islet response. This is both reassuring and of physiological relevance, since preserving β-cell function is fundamental to maintaining optimal glycemic control (24), and augmenting an appropriate early insulin secretory response plays a major role in PPG regulation (25).

Despite enhancing β-cell function, overall insulin secretion was unaffected by our WP preload. Instead, our data indicate that pre-meal WP affects insulin kinetics, namely by reducing MCRi. This observation was intriguing, particularly since the decline in postprandial insulin clearance appears blunted in T2D (12), as was demonstrated here during the placebo trial. By contrast, we found that pre-meal WP augmented a marked decline in MCRi from fasting values, and when compared with the placebo trial, overall MCRi was ∼22% lower. Since circulating plasma insulin concentrations reflect the balance between MCRi and ISR, the observed reduction in MCRi during the WP trial would have enabled more insulin to reach the periphery. As such, our data imply that the predominant contributor to the increase in plasma insulin commonly reported following pre-meal WP (21–23) is due to a reduction in MCRi and not an increase in ISR. The latter finding was in contrast to our initial hypothesis and was surprising given the marked secretion of the incretin peptide, GLP-1 (26), and the fact that protein-stimulated insulin release remains intact in people with T2D (27). Whether the increased secretion of GLP-1 or the increased delivery of amino acids to the liver affected insulin clearance and extraction in the current trial remains unclear, though recent work has highlighted a potential role of endogenous GLP-1 in the regulation of hepatic insulin clearance (28).

Our findings concerning the role of MCRi and ISR on PPG homeostasis following a WP preload are novel and may be of interest in the pathogenesis of T2D. As was demonstrated here, for individuals with compromised β-cell function, a reduction in postprandial MCRi enabled an appropriate insulin profile to be achieved without placing further stress on the β-cells for insulin release. This subsequently resulted in a 16% reduction in PPG iAUC, which may concomitantly improve β-cell viability through a reduction in glucotoxicity within the β-cell (29, 30). In this regard, and where insulin secretagogue therapies may accelerate the decline in β-cell function (2), and increased MCRi are associated with a steeper trajectory of worsening HbA_1c_ (13), we believe these findings are of interest. Clearly, longer-term investigations studying the effects of mealtime WP supplementation on β-cell functionality are warranted.

While the mechanism(s) explaining the observed reduction in MCRi are unclear, explorative analysis highlighted an inverse relationship between glucagon iAUC_240min_ and MCRi (*r_s_* = −0.393; *P* = .018). Thus, a reduction in MCRi, thereby increasing peripheral insulin availability, may mitigate glucagon's actions on hepatic glucose output by facilitating peripheral glucose uptake. However, to the best of our knowledge, this is the first observation of an association between glucagon secretion and MCRi, with previous work in mice (31) and healthy humans (32) failing to report an effect of exogenous glucagon on insulin clearance. Nevertheless, and in support of our hypothesis, bidirectional changes in MCRi and basal hepatic glucose output have been reported during a hyperinsulinemic-euglycemic clamp, independent from any changes in peripheral glucose tolerance (33). Similar findings have also been reported when studying the diurnal responses to feeding in healthy adults. In the latter, although endogenous glucose production and glucagon secretion were less suppressed at a breakfast meal compared with identical meals consumed later in the day, PPG excursions were found to be lower due to an increase in β-cell glucose sensitivity and a reduction in MCRi (34). Unfortunately, tracing PPG fluxes were outside the scope of the present study and, as such, future work is required to provide further insight into the intricacies of PPG handling after pre-meal WP.

As anticipated, pre-meal WP augmented the early and sustained increase in GLP-1 secretion, which most likely reflects the rapid delivery of amino acids to the small intestine and the subsequent depolarization of the enteroendocrine L-cell (35). GIP concentrations were also increased during the latter stages of the trial; though GIP's contribution to the observed glucose-lowering was likely minor since endogenous GIP has little to no effect on postprandial glucoregulation in T2D (36). On the other hand, the glucose-lowering efficacy of endogenous GLP-1 remains partly intact (37). Although the current study cannot delineate the exact role of the WP-mediated increase in GLP-1 to the observed reductions in PPG, it appears that gastric emptying may have been delayed, which is consistent with previous reports (22, 23). This was evident when observing the postprandial responses of GIP, where despite the early elevation in GIP during the WP trial, GIP concentrations were reduced at *t* = 30 minutes, indicating that the transit of nutrients from the stomach to the duodenum were delayed (38, 39). As previously mentioned, the increase in GLP-1 may have also contributed to the reduction in MCRi (28) and enhancement in β-cell function (40).

Strengths associated with our study include the randomized, placebo-controlled design and counterbalanced administration of treatments. Moreover, prehepatic ISR were calculated, thus circumventing interindividual hepatic and posthepatic insulin extraction. We also used a conventional mixed-nutrient meal to convey the expected islet and gastrointestinal responses to the ingestion of a commonly consumed meal, thereby increasing the real-world validity of our findings. Similarly, we chose to present our pre-meal shots at a time that was deemed more likely to embody free-living eating patterns. Nevertheless, it must be recognized that the reported data were secondary outcomes from our primary study (6) and our analyses were conducted on a small sample size (n = 18), though our randomized controlled, crossover study design would have reduced any potential confounders to our findings. Additionally, our modeling analyses are dependent on the assumption that the clearance of C-peptide is constant (17) and is unaffected by a low dose of WP. However, it is unlikely that, in a complication-free cohort of T2D, 15 g of WP would affect glomerular filtration rate and C-peptide kinetics of patients (41, 42).

In summary, the ingestion of a low dose of pre-meal WP reduces PPG excursions in adults with T2D by coordinating an enhancement in β-cell function with a reduction in MCRi to produce an efficient plasma insulin profile. The reduction in insulin clearance may represent a counterregulatory response to an increase in glucagon signaling and hepatic glucose output. Studies tracing PPG fluxes with appropriate methodology following pre-meal WP warrant future evaluation.

## Data Availability

The raw data supporting the conclusions of this article will be made available upon reasonable request.

## Abbreviations

ELISA: enzyme-linked immunosorbent assay
GIP: glucose-dependent insulinotropic polypeptide
GLP-1: glucagon-like peptide 1
HbA_1c_: glycated hemoglobin
iAUC: incremental area under the curve
ISR: insulin secretion rate
MCRi: metabolic clearance rate of insulin
OGIS: oral glucose insulin sensitivity index
PPG: postprandial glycemia
Rdins(t): rate of insulin extraction
T2D: type 2 diabetes
Vins: volume distribution for insulin
WP: whey protein
